# Plasticity in Pro- and Anti-tumor Activity of Neutrophils: Shifting the Balance

**DOI:** 10.3389/fimmu.2020.02100

**Published:** 2020-09-02

**Authors:** Charita Furumaya, Paula Martinez-Sanz, Panagiota Bouti, Taco W. Kuijpers, Hanke L. Matlung

**Affiliations:** ^1^Department of Blood Cell Research, Sanquin Research, Amsterdam University Medical Center, University of Amsterdam, Amsterdam, Netherlands; ^2^Department of Pediatric Immunology, Rheumatology and Infectious Diseases, Emma Children’s Hospital, Amsterdam University Medical Center, University of Amsterdam, Amsterdam, Netherlands

**Keywords:** neutrophils, cancer, tumor microenvironment, myeloid-derived suppressor cells, antibody-dependent cellular cytotoxicity, antibody therapy

## Abstract

Over the last decades, cancer immunotherapies such as checkpoint blockade and adoptive T cell transfer have been a game changer in many aspects and have improved the treatment for various malignancies considerably. Despite the clinical success of harnessing the adaptive immunity to combat the tumor, the benefits of immunotherapy are still limited to a subset of patients and cancer types. In recent years, neutrophils, the most abundant circulating leukocytes, have emerged as promising targets for anti-cancer therapies. Traditionally regarded as the first line of defense against infections, neutrophils are increasingly recognized as critical players during cancer progression. Evidence shows the functional plasticity of neutrophils in the tumor microenvironment, allowing neutrophils to exert either pro-tumor or anti-tumor effects. This review describes the tumor-promoting roles of neutrophils, focusing on their myeloid-derived suppressor cell activity, as well as their role in tumor elimination, exerted mainly via antibody-dependent cellular cytotoxicity. We will discuss potential approaches to therapeutically target neutrophils in cancer. These include strategies in humans to either silence the pro-tumor activity of neutrophils, or to activate or enhance their anti-tumor functions. Redirecting neutrophils seems a promising approach to harness innate immunity to improve treatment for cancer patients.

## Introduction

The new immunotherapies aimed at targeting of immunosuppressive checkpoint receptors, using antibodies against the inhibitory programmed cell death protein 1 (PD-1)/programmed death-ligand 1 (PD-L1) pathway or cytotoxic T-lymphocyte-associated protein 4 (CTLA-4) pathway, have vigorously changed the landscape of anti-cancer immunotherapy in the last decades (1). This way of harnessing capacities of the immune system to combat cancer has recently delivered the first unprecedented clinical successes in the treatment of difficult-to-treat cancers, such as advanced stage metastatic melanoma (2), and non-small cell lung carcinoma (3), as well as more recently in other cancer types (4–6). Despite the encouraging efficacy seen for some cancer patients, where durable responses are observed for several years, others either fail to respond to these therapies or acquire resistance over time (7). Another recent and successful approach of cancer immunotherapy consists of the use of modified T cells with chimeric antigen receptors, the so-called CAR T cells. These have shown promising results for patients with advanced B-cell cancers in the early days (8) and more recently also in the case of other previously incurable malignancies, due to their specificity for a cell-surface antigen (9). Yet, these novel therapies still raise certain general concerns to clinicians, as they have been associated with serious toxicities (10). Although the current focus of cancer immunotherapies is on targeting adaptive immunity to fight the tumor, these therapies are limited to specific cancer types or need more efficient therapeutic control to reduce adverse effects. Besides, the cytotoxic anti-tumor capacities, as well as CAR T cell expansion, can easily be hampered by the interaction with other immune cells present in the tumor microenvironment (TME).

A different approach to immunotherapy in cancer focuses on the innate immune system. Cells belonging to the innate compartment are endowed with the capacity of reacting fast to invading pathogens that enter the body by recognizing sets of repeated patterns, as well as damage signals released from tissue injuries. In this way, innate immune cells have the outstanding talent of distinguishing self from non-self and respond to the latter in an appropriate manner. In addition, such cells are also able to distinguish self from “altered-self,” and this is the case for cancer. Innate immune cells such as natural killer (NK) cells, dendritic cells (DC), neutrophils, and macrophages are first-line effectors for the elimination of cancer cells before an adaptive immune response is mounted. In this way, NK cell-based therapies (i.e., used in the form of adoptive cell transfer or as CAR NK cells) (11), as well as DC-based therapies (i.e., used for vaccination against cancer) (12, 13) are currently being exploited for the treatment of cancer. However, the clinical application and efficacy of these therapies can be hampered as these approaches are still in early stages of development. Alternatively, neutrophils or polymorphonuclear (PMN) leukocytes, as the most abundant cell type among the circulating white blood cells in humans, can also be considered as compelling cells to address therapeutically.

## Neutrophils: From Innate Effector to Myeloid Suppressor Cells

Neutrophils are commonly short-lived cells with a half-life in the circulation of less than 24 h and one or more days in the tissues depending on the extravascular milieu. Thus these cells are renewed in the bone marrow at an estimated rate of 10^11^ cells per day. The percentage of neutrophils in blood in healthy adults ranges from 50 to 70% (14), although these numbers may differ under pathological conditions (15). The mobilization of neutrophils from the bone marrow into the circulation is defined by the secretion of various stimuli from the site of injury or disease, to which they respond, resulting in variations in neutrophil numbers in blood. For example, granulocyte-colony stimulating factor (G-CSF), which is frequently produced by certain tumors or by cell types surrounding the tumor (16, 17), is able to skew the neutrophil retention/release balance in the bone marrow and ultimately lead to an increased release of neutrophils into the circulation (18). Moreover, chemokines, such as IL-8, can also be produced by tumor cells and attract myeloid cells, including neutrophils, to the TME, thereby affecting the number of neutrophils in the tumor (19). To date, many studies have looked at the neutrophil-to-lymphocyte ratio (NLR) to determine the correlation between the number of circulating neutrophils and cancer prognosis. In all cases, a high NLR has been recognized as a bad prognostic marker for all cancer types and stages of cancer (20–22). Moreover, the extent of intra-tumoral neutrophils also seems to have an unfavorable prognostic value in several cancer types (23–25). On the other hand, low neutrophil counts in blood has been proven advantageous for survival (26). Nonetheless, the option of persistent neutrophil depletion cannot be regarded in the context of cancer since prolonged neutropenia is considered a life-threatening condition due to the indispensable role of neutrophils in the protection of the host in the natural (mucosal) barriers against incoming microbial pathogens.

In fact, neutrophils in cancer may represent a heterogeneous population of cells, which can display different phenotypes and perform opposing functions. In mice, neutrophils found in tumor tissue have been referred to as tumor-associated neutrophils (TANs), which were classified as either anti-tumor neutrophils (N1), or tumor-promoting neutrophils (N2) (27). In experimental models, several factors of the TME have been suggested to determine the polarization of the recruited neutrophils in the tumor toward one or the other phenotype *in vivo*. Supportive evidence of such neutrophil subsets in human cancer tissue is, however, lacking to date. Studies in cancer patients have reported that circulating neutrophils can be classified into different subpopulations according to their densities upon isolation and centrifugation. Normal or high density neutrophils (HDNs) are associated with anti-tumor activity, whereas neutrophils in the low-density fraction (LDNs) are believed to expand in malignancy and display myeloid-derived suppressor cell (MDSC) and other pro-tumor activity. Similar to TANs, HDNs are capable of switching to LDNs in response to factors in the surrounding environment (28). Unfortunately, the current understanding of neutrophil subpopulations is still limited and debated due to the lack of specific molecular markers, non-uniform study approaches and variable expertise. Nonetheless, it is clear that neutrophils are being increasingly recognized as important players in cancer, and that they can carry both pro- and anti-tumoral properties depending on cancer type, stage and location of the disease (29).

In the following chapters, we aim to provide a summary of the current knowledge and latest findings on pro- and anti-tumor activities of neutrophils by describing the main mechanisms of action and highlight some conceivable ways by which we could silence tumor-promoting activity or redirect the activity of pro-tumor neutrophils into cytotoxic effector cells to help combat cancer.

## Role of Neutrophils in Tumor Progression

### Promotion of Proliferation, Angiogenesis, Invasion and Metastasis by Neutrophils

Neutrophils are capable of promoting tumor growth and progression, and their presence is often associated with poor clinical outcome (20). Mechanisms by which neutrophils have been shown to mediate tumor progression include enhancing proliferation, angiogenesis, invasion, metastasis, and immune suppression (30). Neutrophils in the TME have the ability to directly induce the proliferation of cancer cells, for example via the serine protease neutrophil elastase (NE) (31, 32). NE also plays a role in the migration and invasion of cancer cells (33). Other neutrophil granule components such as matrix metalloproteinase-9 (MMP-9) have been described to mediate angiogenesis and tumor cell invasion via degradation of the basement membrane (34). Additionally, a large body of literature demonstrates a pro-metastatic function of neutrophils. In a mouse breast cancer model, neutrophil-derived factors were shown to drive cancer spread (35). Furthermore, neutrophils have been suggested to promote cancer cell adherence, which was shown to be dependent on neutrophil Mac-1 (αMβ2 or CD11b/CD18), and thereby mediate metastasis in a murine model of liver metastasis (36). Concordantly, human neutrophils were shown to induce tumor cell migration and to interact with melanoma cells via β2 integrin (37).

Also the involvement of neutrophil extracellular traps (NETs) in cancer cell migration and extravasation is being investigated. Upon activation, neutrophils form NETs composed of released chromatin and granular proteins which trap and kill microbes (38). The neutrophil chemoattractant IL-8, which is produced in the TME, has been suggested to induce NET formation (39). The presence of NETs in the TME of patients with metastatic disease has been demonstrated, and additional studies in murine models have further suggested their role in cancer progression (40). NETs promoted cancer cell migration, invasion, and angiogenesis *in vitro* (41). Multiple studies illustrated the trapping of circulating murine tumor cells in NETs, which facilitated their extravasation and metastasis (42–44). Increased levels of NETs were also observed in patients suffering from different types of locally infiltrating cancer (45, 46), which was associated with adverse patient outcomes in colorectal cancer (47).

### Immunosuppression by Neutrophils

In mice, MDSCs represent a heterogeneous group of pathologically activated immature myeloid cells with immunosuppressive properties (48). MDSCs accumulate under inflammatory conditions, including experimental cancer, and are divided into two major subsets depending on their lineage, either granulocytic (PMN-MDSCs) or monocytic (M-MDSCs) (49). The presence of PMN-MDSCs in patients has been shown to be associated with poor prognosis in different types of cancer (50–52). In mice, PMN-MDSCs are characterized as CD11b^+^Ly6G^+^ cells, while in humans the surface marker definition is CD11b^+^CD15^+^CD14^–^CD33^+^CD66b^+^HLA-DR^–^ (53). However, based on these cell surface markers, PMN-MDSCs overlap with all circulating neutrophils, making an accurate discrimination between PMN-MDSCs and neutrophils impossible. Also other markers proposed to be more specific in identifying PMN-MDSCs, such as LOX-1 or CD10 (54, 55), have not been confirmed to discriminate circulating PMN-MDSCs in cancer patients (56).

While PMN-MDSCs were originally described as a subpopulation of immature myeloid cells capable of suppressing immune responses, mature neutrophils also have the ability to limit T cell activity and promote immune evasion (28, 57), but only upon cellular activation (56, 58). Thus the functional similarities between PMN-MDSCs and neutrophils further complicate the differentiation between the two populations. Functional plasticity of neutrophils suggests that a shift in neutrophil phenotype occurs, depending on signals from the TME, which lead to the acquisition of immunosuppressive activity or other pro-tumorigenic functions. To avoid confusion, we will mostly refer to these cells as immunosuppressive neutrophils. Such mature neutrophils with a T cell suppressive phenotype have been identified in various human cancers and are also associated with accelerated tumor progression and worse clinical outcomes (49, 58), illustrating their clinical relevance as potential targets to improve cancer immunotherapy.

#### Activation of Neutrophil Immunosuppressive Activity

Tumor cells and other cell types in the TME produce a wide range of inflammatory mediators, many of which have been demonstrated to contribute to the generation and recruitment of neutrophils with pro-tumor activity. High levels of the colony stimulating factor G-CSF released by tumors corresponds with the expansion of immunosuppressive neutrophils in cancer patients (50). Likewise, mature neutrophils of G-CSF-treated donors have been reported to display an activated immunosuppressive phenotype (55). Other signals implicated in the pathological activation of neutrophils include GM-CSF, TNFα, IL-1β, VEGF, IL-6, and IL-8 (59). However, our latest experiments in human neutrophils demonstrated that only fMLF, TLR ligands such as LPS, and TNFα act as activators of T cell suppressive activity in neutrophils (56, 60). The presence of soluble factors in ascites and malignant effusions from cancer patients was shown to induce a suppressive phenotype of neutrophils in the TME, which was dependent on complement factor C3 (58).

#### Mechanisms of Neutrophil Immunosuppressive Activity

In order to limit T cell mediated anti-tumor immune responses, suppressive neutrophils rely on several effector functions originally linked to their role as killers of invading pathogens. Degranulation refers to the process by which neutrophils release various factors stored in intracellular granules into phagosomes or the extracellular environment (61). Immunosuppression by neutrophils has been linked to the metabolism of L-arginine, which is converted into L-ornithine by arginase-1, an enzyme present in gelatinase granules (62, 63). Elevated arginase-1 plasma levels were observed in cancer patients, and the modulation of T cell responses was shown to be dependent on arginase-1 (50, 64, 65) via the depletion of L-arginine, an amino acid crucial for the expression of the T cell receptor ζ chain, which is in turn needed for T cell activation (66–68). Additionally, L-arginine shortage prevents the successful formation of immunological synapses due to impaired dephosphorylation of cofilin, which is an important player in the modulation of the actin cytoskeleton and the formation of an immunological synapse (69, 70). The dependence on arginase-1 and its regulation of the T cell receptor ζ chain was demonstrated using PBMCs from healthy donors (71, 72) and in cancer patients (64, 73). Accordingly, suppression of T cell mediated responses by activated human neutrophils was shown to depend on degranulation, which was elegantly confirmed using neutrophils from rare familial hemophagocytic lymphohistiocytosis (FHL)-5 patients, which show defective granule mobilization due to mutations in the *STXBP2* gene (56, 74–76).

The nicotinamide adenine dinucleotide phosphate (NADPH) oxidase complex generates oxidative stress by the production of reactive oxygen species (ROS) upon cellular activation, a process which is up regulated in different types of cancer (77). Hydrogen peroxide (H_2_O_2_) produced by the conversion of superoxide (O_2_^–^) suppresses T cell activation and proliferation via several mechanisms (78, 79). H_2_O_2_ can induce T cell apoptosis, or inhibit T cell activation by blocking NF-κB activation or affecting the availability of the T cell receptor ζ chain, which is essential for T cell activation, as described above (80, 81). T cell activation is additionally impaired by the interference of ROS with the regulation of cofilin (82, 83), as was also described as an effect of L-arginine deficiency. While L-arginine deficiency leads to impaired dephosphorylation of cofilin, ROS induce a conformational change in cofilin through its oxidation, both resulting in an inhibition of T cell response (69, 82, 83). Furthermore, H_2_O_2_ could be involved in preventing the metabolic switch from mitochondrial respiration to aerobic glycolysis that is required for T cell clonal expansion and cytokine production (84). Our data also indicate that ROS production is required for the suppression of T cell activation by human neutrophils (56). This was substantiated using neutrophils from patients with chronic granulomatous disease (CGD), a rare genetic defect in which neutrophils are incapable of ROS production due to a mutation in a subunit of the NADPH oxidase complex (85, 86).

T cell function could additionally be impaired by suppressive neutrophils via the interaction between T cell PD-1 and PD-L1 on neutrophils, which is known to block T cell proliferation and cytokine production (87). Crosstalk between PD-1 and PD-L1 plays an important role in T cell suppression in cancer (88). Neutrophil expression of the immune checkpoint surface molecule PD-L1 can be induced by IFNγ and GM-CSF or hypoxic conditions (89–91). Increased PD-L1 expression as well as suppression of T cell proliferation by neutrophils were demonstrated in patients with hepatocellular carcinoma (92). Moreover, inhibition of T cell function by neutrophils was dependent on PD-1 expression by T cells and PD-L1 expression by neutrophils. PD-L1^+^ neutrophils suppress T cell function by interacting with PD-1 on T cells, thus limiting the anti-tumor activity of T cells. This immune tolerance via neutrophil PD-L1 appears to contribute to human gastric cancer growth and progression (93).

In order for neutrophils to suppress T cell responses, the formation of close cell-cell contact via the expression of integrin Mac-1 on neutrophils is required (94). Singel et al. recently demonstrated the requirement of cell-cell contact for T cell suppression by human neutrophils activated by ovarian cancer ascites (58). This is in line with our findings, which demonstrate that suppression of T cells by neutrophils is dependent on CD11b-mediated interactions (56). Consistently, neutrophils from a patient with leukocyte adhesion deficiency type 1 (LAD-1), which lack the expression of β2 integrins, including Mac-1, were not capable of suppressing T cell proliferation (56). Blocking ICAM-1 on T cells decreased, but did not fully abrogate T cell suppression by activated neutrophils, indicating that additional ligands of Mac-1 on T cells are important for the interaction with neutrophils (56). In tumor-bearing mice, neutrophils were shown to induce T cell apoptosis in a contact-dependent manner (95). Similarly, an immunosuppressive myeloid cell population induced apoptosis of activated T cells in breast cancer patients (96). In our studies, live-cell imaging revealed intercellular contacts between neutrophils and T cells resulting in neutrophils containing pieces of T cell membrane (56), a process known as trogocytosis (97). During co-culture of activated neutrophils and T cells, a population of smaller T cells appeared, which could no longer be activated to proliferate. In contrast to earlier findings with respect to apoptosis induction, these small T cells displayed neither of the two common cell death mechanisms, i.e., apoptosis or necroptosis. Thus, the exact steps leading to T cell death upon suppression by neutrophils remain to be further investigated.

In addition to their own direct and suppressive effect on T cells, neutrophils can also inhibit NK cell activation in tumor-bearing mice (98, 99). Neutrophils suppressed NK cell cytotoxicity, which resulted in defective antitumor responses and promoted metastasis in mice (100, 101). Finally, further contribution of neutrophils to the immunosuppressive microenvironment within tumor tissue is made via the development and induction of regulatory T cells (Tregs) (102). Treg enrichment occurs due to reduced sensitivity of Tregs to oxidative stress in the TME (103, 104), which ultimately accounts for their preferential outgrowth and selection. Neutrophils also recruit Tregs through the secretion of CCL17, as was shown in murine tumors (105). Additionally, a positive feedback loop between neutrophils and Tregs, mediated by the anti-inflammatory cytokine IL-10, could support the immune suppression (106, 107). Induction of Tregs by suppressive neutrophils was demonstrated in patients with bladder cancer (108).

In summary, neutrophils can directly and indirectly mediate tumor progression via the promotion of proliferation, angiogenesis, invasion, and metastasis, in which mechanisms such as the release of granule components and NET formation are important. Furthermore, neutrophils inhibit anti-tumor responses by limiting T cell activation, which is dependent on degranulation, ROS production and cell-cell contact. Alternatively, neutrophils contribute to an immunosuppressive TME by affecting other immune cells, such as NK cells and Tregs. Apart from the pro-tumorigenic mechanisms by which neutrophils are activated to “adopt” their tumor-enhancing, immunosuppressive role, neutrophils can also exert strong anti-tumor activity.

## Role of Neutrophils in Tumor Elimination

The vast majority of studies support the positive correlation of neutrophils with cancer progression by the mechanisms described above. Nevertheless, recent data also point into the opposite direction in which neutrophils can act as effector cells and combat cancer leading to the eradication of tumor cells (30, 109, 110). In fact, there are sufficient reasons to think of neutrophils as promising effector cells against cancer. One of the main advantages of this cell type is that they are found in high levels in blood under normal conditions, and in addition, their number can be incremented further upon *in vivo* treatment with G-CSF and GM-CSF cytokines (111, 112). Another feature that makes them unique is that neutrophils do not need *ex vivo* culturing and expansion for a later infusion into the patient, compared to most other immune cells used therapeutically, such as T cells, NK cells or DCs (113). Siders et al. showed that increasing the number of circulating neutrophils by a mere injection of G-CSF turned them into excellent cytotoxic cells that were able to kill alemtuzumab-opsonized cells in a xenograft model of a CD52^+^ tumor (114). This, however, must be regarded with caution as such effect may only be valid in the context of antibody therapy, as was also shown in other studies (115, 116). In other circumstances, the therapeutic effect of G-CSF treatment may mainly mobilize neutrophils of an immunosuppressive phenotype which are beneficial to the tumor, as discussed earlier in this review (50, 55).

### Direct Killing Properties of Neutrophils Against Cancer

The mechanisms by which anti-tumor neutrophils induce cytotoxicity of the tumor cells are, however, not yet clear. Few studies demonstrated that the secretion of ROS through the respiratory burst comes into play once neutrophils are in direct contact with the tumor cells (117–119). Specifically, neutrophil secretion of H_2_O_2_ induced a lethal influx of Ca^2+^, mediated by the TRMP2 channel which ultimately killed the tumor cell. Furthermore, a mechanism was identified by which neutrophils isolated from healthy donors induced apoptotic cell death of tumor cells upon physical contact mediated through Fas ligand/Fas interaction (120). Although these contact-dependent processes can, under specific circumstances, induce tumor cell killing, the best well-established anti-tumor mechanism by which neutrophils mediate tumor cell death is through Fc receptor-dependent cytotoxicity against antibody-opsonized cells.

### Antibody-Mediated Killing Properties of Neutrophils Against Cancer

Therapeutic monoclonal antibodies (mAbs) have become available for the treatment of cancer in the last decades and have provided significant improvement in the treatment outcome for a number of cancer subtypes. These mAbs can initiate direct tumor cell killing, through the F(ab’)_2_ domains of the immunoglobulin (Ig) interfering with the intrinsic function of the target cell (121, 122), or upon binding of the Fc part to the C1q component of the complement system inducing complement-dependent cytotoxicity (CDC) (123). Besides the direct mechanisms, the Fc region of the mAb can also bind to activating Fc receptors on innate immune cells which will in turn elicit indirect-mediated killing via antibody-dependent cellular cytotoxicity/phagocytosis (ADCC/P) of the opsonized cells (124–128).

The contribution of neutrophils in ADCC to eradicate antibody-opsonized tumor cells *in vitro* was already demonstrated some decades ago by Gale and Zighelboim (129). A few years later, Barker and colleagues showed a prominent role of neutrophils isolated from neuroblastoma patients in mediating the *ex vivo* lysis of neuroblastoma cells opsonized with anti-GD2 antibody. Interestingly, the cytotoxicity induced by neutrophils was in all cases higher than was seen for NK cells from the same donor (130). More recently, a number of studies have demonstrated the cytotoxic capacity of neutrophils in the context of antibody therapy in an *in vivo* setting of tumor-bearing mice which showed once again the indispensable role of neutrophils in achieving efficient antibody therapy responses (114, 131, 132). Most importantly, a considerable amount of literature has suggested the relevance of the anti-tumor capacities of neutrophils in a clinical setting. More specifically, different polymorphic variants found on the IgG Fc receptor IIa (FcγRIIa) explained the variability seen in the clinical outcome of breast cancer (133, 134), colorectal cancer (135), and neuroblastoma patients (136) in response to antibody treatment.

#### IgG Isotypes and Fcγ Receptor Involvement

Despite the fact that several Ig isotypes exist, as of yet, all approved therapeutic antibodies for cancer treatment in the clinic are IgG-based, particularly IgG1, IgG2, and IgG4 isotypes. The relevance of the IgG backbone used for such antibodies has to do with the different binding affinities to the several Fcγ receptors that are in turn differentially expressed on a number of immune cells (137, 138). This results in the induction of extremely diverse and highly regulated antibody responses, as the distinct affinities can also convey stronger or weaker effector functions by the different Fcγ receptor-expressing cells (139).

Six classical Fcγ receptors can be expressed by the human innate immune cells: FcγRI (CD64), FcγRIIa (CD32a), FcγRIIb (CD32b), FcγRIIc (CD32c), FcγRIIIa (CD16a), and FcγRIIIb (CD16b). From these, neutrophils constitutively express FcγRIIa and FcγRIIIb, and the FcγRI only upon activation. Depending on ethnic background, 15% of the population also expresses low levels of neutrophil FcγRIIc (140). All these Fcγ receptors on neutrophils can bind to the IgG opsonizing cancer cells (138, 141) and have their own capacity to contribute to ADCC activity. FcγRI and FcγRIIa/c are activating receptors and their signals are transduced by an immunoreceptor tyrosine-based activation motif (ITAM) that is either associated with the common FcRγ chain in the case of FcγRI, or present in the cytoplasmic tail of the Fcγ receptor itself for FcγRIIa/c. Subsequently, Syk tyrosine kinases can bind to these activating motifs and activate downstream signaling pathways, initiating ADCC, phagocytosis, cell migration, and degranulation processes (141, 142). FcγRIIIb, instead, is anchored to the cell membrane through a glycosyl phosphatidylinositol (GPI) molecule and lacks both transmembrane and cytoplasmic domains, restricting it from having signaling capacities (143). As mentioned above, resting neutrophils constitutively express both low- and intermediate- affinity FcγRIIa and FcγRIIIb in high levels (141). However, the high-affinity activating FcγRI is not detectable on resting neutrophils but can be up regulated upon activation of cells with G-CSF and IFNγ as demonstrated by us (30, 144) and others (145, 146). In addition, FcγRIIIb is actively shed from the surface of activated neutrophils when stimulated by various stimuli, including G-CSF and IFNγ (144, 147).

The specific contribution of each Fcγ receptor of neutrophils to mediate ADCC, however, has been found to differ per cancer type. With the use of blocking antibodies for the different Fcγ receptors, several studies have reported FcγRIIa to be the dominant receptor triggering neutrophil ADCC in solid tumors. This was seen for EGFR^+^ cancer cells opsonized with cetuximab (148), as well as for trastuzumab-coated HER2/neu^+^ human breast cancer SKBR3 cells (144, 149). Of interest, although FcγRI is the high-affinity receptor for IgG1 antibodies, no effect was found when monovalent Fc fragments were used for blockade. This might be explained by the incapacity to fully block the receptor by use of these monovalent Fc fragments or due to the relatively low expression levels on the surface of neutrophils (150). Nevertheless, contradicting results were described regarding the potential of FcγRI on neutrophils in mediating ADCC. It was shown that such receptor can very well contribute to tumor killing in a number of tumor types (151–154). With regard to the involvement of FcγRIIIb in the killing of tumor cells in the context of antibody therapy, we and others have shown this receptor to be a negative regulator of neutrophil ADCC as it scavenges available therapeutic antibody due to its high expression on the neutrophil surface (144, 148). For hematological tumors the evidence is slightly different. An *in vivo* study testing the efficacy of a FcγRI bispecific antibody in a mouse model of a rituximab-treated B-cell lymphoma reported the clearance of the tumor cells by G-CSF stimulated neutrophils (155), indicating that FcγRI may be the main receptor mediating neutrophil ADCC in such context. Another level of complexity of this receptor family comes from the several Fcγ receptor polymorphisms described in humans that could affect the degree of ADCC responses [extensively reviewed by Bruhns and Jonsson (138)], which ultimately can influence clinical responses to antibody therapy. It is, however, relevant to acknowledge that Fcγ receptors are not the only feature that makes neutrophils capable of killing cancer cells. Although falling outside the scope of this review, integrins, Mac-1 in particular, have also been shown to be indispensable in mediating ADCC processes (149).

#### Neutrophil-Mediated Antibody-Dependent Killing Mechanisms

Until recently, the mechanism by which ADCC leads to cell death remained largely unclear. A large body of evidence has reported that neutrophils have the ability to trogocytose, mainly in the context of antibody therapy (156). In 2002, trogocytosis was identified as an active mechanism that involves the transfer of plasma membrane and their associated molecules from a donor cell to an acceptor cell during intercellular contact (157). Although the purpose of the process has remained unclear (97), more recently, new evidence has accumulated to determine the importance of trogocytosis. In the context of infection, neutrophils were able to kill serum-opsonized *Trichomonas vaginalis* parasite using trogocytosis (158). Additionally, the tumoricidal effect of trogocytosis was shown in the study of Velmurugan et al., where they demonstrated that macrophage-trogocytosis led to efficient tumor cell death of trastuzumab-opsonized breast cancer cells (159). Most recently, we reported a direct association between neutrophil-mediated trogocytosis and tumor cell killing in antibody-opsonized solid cancer cells (149), by which the neutrophil takes “bites” from the plasma membrane of the cancer cells. Hereby, the neutrophils cause membrane damage eventually leading to a necrotic type of cancer cell death. Furthermore, neutrophil-mediated antibody-dependent destruction of cancer cells was found not to depend on their classic antimicrobial effector mechanisms, such as granule exocytosis (degranulation) and NADPH oxidase activity. In particular, neutrophils from FHL-5 patients, as well as neutrophils from patients with CGD, showed unexpectedly intact killing of HER2/neu^+^ breast cancer cells in the presence of the therapeutic antibody trastuzumab (149). Together, these findings support the idea of trogocytosis as a most relevant process involved in tumor killing in the context of antibody therapy.

Conversely, trogocytosis has also been described as a mechanism to escape ADCC, ADCP, or CDC, mainly in hematological cancers, as it involves the shaving of the target antigen from tumor cells by the effector cells. This was shown not only for chronic lymphocytic leukemia (CLL) upon rituximab treatment, where a partial loss of CD20 from CLL B cells accompanied trogocytosis events by neutrophils (160), but also anti-CD38 therapy with daratumumab directed against multiple myeloma (MM) which led to monocyte- and neutrophil-mediated shaving of CD38 from MM cells (161). Moreover, neutrophil-mediated trogocytosis might be the reason behind the significant reduction in HER2/neu expression, seen in a cohort of breast cancer in women treated with trastuzumab (162, 163). Therefore, depending on the circumstances, or perhaps tumor type, trogocytosis can be regarded either as a process initiating tumor killing or as a way for the tumor cells to evade immune activity.

### Role of Neutrophils Regulating Adaptive Immune Response

For cancer therapy, it has now become clear that initiating potent adaptive immune responses is fundamental to establish long-term anti-tumor immunity. Although neutrophils have historically been regarded as strict innate cells with end-stage effector functions, new evidence has emerged manifesting their involvement in modulating the adaptive immune compartment (31, 32). At sites of infection, neutrophils were found to act as danger sensors by communicating the presence of inflammation or damage to DCs, which induces DC maturation, triggering in turn strong proliferation and T_*h*_1 polarization of naive CD4^+^ T cells (164, 165). In addition, neutrophils can also act as APCs themselves. *In vitro*, activated neutrophils stimulated with GM-CSF and IFNγ were able to present antigens to memory CD4^+^ T cells due to the acquisition of MHC-II molecule expression, as well as costimulatory molecules such as CD86, OX40L and 4-1BBL at early stages of tumorigenesis (166–168). Moreover, both human and mouse neutrophils were found to cross-present exogenous antigens to naive CD8^+^ T cells thereby turning them into cytotoxic T cells (169). Lastly, recent evidence suggests that upon antigen capture at the periphery, neutrophils can migrate to the lymph nodes in a CC-chemokine receptor 7 (CCR7)-dependent manner, under certain circumstances, as their presence has been found in lymphoid organs *in vivo* (170–172).

In the context of cancer, however, neutrophils can mediate opposite adaptive immune responses depending on the TME or stage of the tumor *in vivo*. For some cancer mouse models, neutrophil depletion led to a decrease in CD4 and CD8 T cell activation, thereby enhancing tumor growth (173), while in others their presence was able to suppress CD8 T cells and promote metastasis (174). The evidence for the role of neutrophils in inducing adaptive immune responses after antibody therapy of cancer is, unfortunately, more scarce. Yet, neutrophils were able to boost T-cell activation when combining a Fc/IL-2+TA99 antibody with adoptive T-cell transfer in a B16F10 melanoma model achieving significant tumor control (175). In general, neutrophils can not only play a role in innate immunity but also guide and support adaptive immune responses through cellular crosstalk.

## Targeting Neutrophil Activity in Cancer Therapy

The different functions that neutrophils acquire in the context of cancer highlight their plasticity and ability to respond toward various targets within and outside of the TME. In this review, we have focused on the role of neutrophils in tumor progression or tumor elimination and we have described the major mechanisms that neutrophils utilize to achieve an efficient response. Although mechanistically not yet completely understood, some reports (28, 176) suggest TANs in several mouse models of cancer to display a gradual change during tumor progression, shifting from anti-tumor properties at the early stages toward pro-tumorigenic properties during the course of the disease.

As reviewed above, both immunosuppressive as well as anti-tumor neutrophils share some common characteristics, i.e., their need for activation through specific stimuli to be able to optimally exert their function (27, 50, 59, 177). Another necessity for neutrophils to perform their MDSC and anti-tumor function is Mac-1-mediated close contact, either between neutrophil and T cell in case of MDSC activity, or between neutrophil and antibody-opsonized tumor cell in case of ADCC (56, 58, 94, 149). At the same time, the fact that classical anti-microbial killing mechanisms, i.e., degranulation and ROS production, are essential for MDSC function of neutrophils, yet dispensable for neutrophil ADCC, shows that at least some of the required cellular mechanisms for MDSC and ADCC neutrophil activity are directly opposing (56, 149).

**FIGURE 1:**
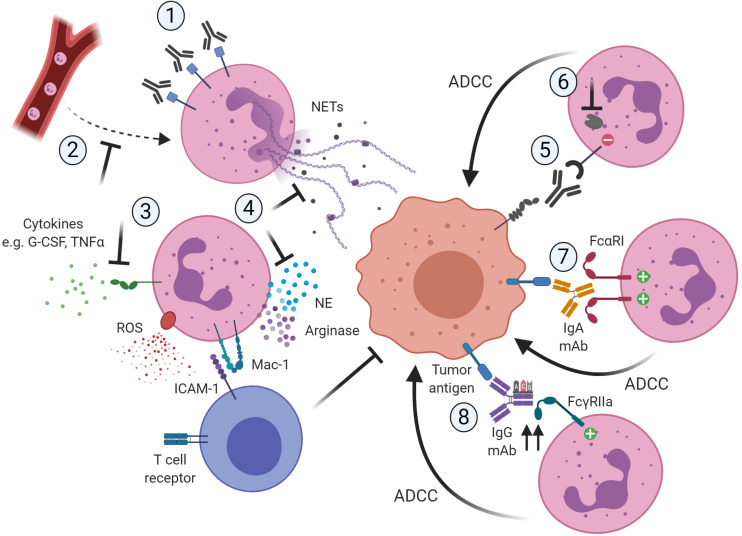
Potential ways to therapeutically target neutrophils in cancer by blocking their pro-tumor activity (1–4) or promoting their anti-tumor capacities (5–8). (1) Reduction of neutrophil numbers in the TME, for example by using antibodies targeting CD33 present on immunosuppressive neutrophils. (2) Blocking the recruitment of pro-tumor neutrophils to the TME, for example by inhibiting the chemokine receptor CXCR2. (3) Blocking of activation signals such as G-CSF or TNFα necessary for neutrophils to acquire a pro-tumor or immunosuppressive phenotype. (4) Neutrophils require ROS production and degranulation to exert their immunosuppressive role, which is dependent on close contact with T cells via Mac-1. Also, neutrophils rely on mechanisms such as NET formation and the release of granule components such as NE to exert their pro-tumor activity. Targeting these downstream mechanisms would limit the pro-tumorigenic activity of neutrophils. (5) Interfering with innate immune inhibitory checkpoints would restore antibody-mediated anti-tumor capabilities of neutrophils. (6) Targeting the recruitment and function of downstream regulators of the inhibitory receptor would further enhance antibody-mediated anti-tumorigenic capacities of neutrophils toward tumor cells. (7) The use of IgA-based therapeutic mAbs that can bivalently bind FcαRI on the neutrophil would induce stronger anti-tumor cytotoxic responses. (8) The use of protein engineering techniques to modify the Fc region of IgG therapeutic antibodies would increase the affinity to the activating FcγRIIa, resulting in more potent ADCC responses toward the opsonized tumor cells. Created with BioRender.com.

Together, in the following part of this review we will elaborate on ways to modulate pro-tumorigenic function, further enhance the anti-tumor response, or even redirect tumor-promoting neutrophils toward anti-tumor neutrophils (Figure 1). Interestingly, the first *in vitro* study showing a way to polarize human neutrophils toward the distinct N1 or N2 phenotype has recently been published (178).

### Limiting Pro-tumorigenic Capacity of Neutrophils

#### Reducing Neutrophil Numbers

The first possibility to interfere with the tumor-promoting role of neutrophils in cancer is the neutralization of these cells. Antibody-mediated depletion of neutrophils resulted in decreased metastasis in an intrasplenic model of liver metastasis and in a metastatic breast cancer mouse model (36, 174). Similarly, targeting of Ly6G^+^ cells in a murine model of pancreatic ductal adenocarcinoma increased intra-tumoral T cell accumulation and inhibited cancer progression (179).

The described promising results of antibody targeting in mice called for a similar approach to be undertaken in humans, in order to promote anti-cancer T cell responses. Based on immunophenotyping and functional assays to detect T cell suppression, the presence of suppressive neutrophils was determined in blood obtained from patients with different types of cancer, including prostate, lung, head and neck, and breast. The immunotoxin Gemtuzumab ozogamicin was used to deplete cells expressing CD33, identified as a surface marker on suppressive cells across cancer types. This depletion restored T cell proliferation, enhanced CAR T cell responses and tumor cell death (180).

Furthermore, a small-molecule receptor tyrosine kinase inhibitor has proven to successfully modulate the immunosuppressive TME. Sunitinib inhibits signaling through multiple receptor tyrosine kinases, including vascular endothelial growth factor receptors (VEGFRs), platelet-derived growth factor receptors (PDGFRα and PDGFRβ), stem cell factor receptor (c-Kit) and colony-stimulating factor-1 receptor (CSF-1R) (181). Treatment with sunitinib decreased the number of suppressive cells, enhanced CD8 and CD4 cell tumor infiltration and improved survival in tumor-bearing mice (182). In renal cell carcinoma patients, sunitinib also reversed T cell suppression and reduced Tregs at the tumor site (183). Moreover, treatment with sunitinib in human renal cell carcinoma improved the expansion of tumor-infiltrating lymphocytes (184).

Since neutrophils were shown to up regulate PD-L1, neutrophils may also be affected by therapies targeting immune checkpoints. For example ipilimumab treatment led to reduced PMN-MDSC numbers and less immunosuppressive activity in melanoma patients, which correlated with improved clinical outcome (185–187). Thus, increasing clinical evidence supports the notion that reducing neutrophil numbers in cancer could be beneficial to patients.

#### Targeting Neutrophil Recruitment and Activation of MDSC Activity

An alternative approach to targeting the pro-tumor activity of neutrophils is to inhibit their recruitment or activation. For instance, IL-8 secreted by tumor cells is responsible for the chemotactic recruitment of neutrophils to the TME via the receptors CXCR1 and CXCR2 (39). Already applied in patients suffering from other inflammatory diseases, inhibition of CXCR2 prevents the recruitment of immunosuppressive neutrophils (188, 189). Recent studies in mouse models of cancer have also shown promising effects of CXCR2 inhibition, which increased effector T cell accumulation in tumors and enhanced responses to immunotherapy, slowing tumorigenesis or preventing metastasis (190–192). The effect of the CXCR1 and CXCR2 inhibitors reparixin and SX-682 are currently being tested in clinical trials in metastatic breast cancer patients (NCT02370238, NCT03161431) (193).

Another example of the stimulation of anti-cancer responses achieved by limiting neutrophil recruitment is the inhibition of the receptor tyrosine kinase cMET (194). cMET is a receptor for hepatocyte growth factor (HGF), which shows increased levels and association with poor clinical outcome in human cancer (195). Limiting neutrophil recruitment by blocking cMET promotes the efficacy of adoptive T cell transfer and checkpoint therapy in murine melanoma (196). Antagonists of the HGF/cMET pathway have been developed and are being tested in multiple types of human cancer (197).

In addition, targeting pathways leading to the pathological activation of neutrophils could represent a strategy to limit their tumor-promoting effects. IL-17 production by γδ T cells was shown to induce G-CSF release in mice, resulting in the accumulation of neutrophils with a T cell-suppressive phenotype (174, 198). In human breast cancer, IL-17 and γδ T cells have also been described as poor prognostic factors (199, 200). Depletion of IL-17, G-CSF or γδ T cells resulted in decreased T cell suppression, and the absence of γδ T cells or neutrophils reduced metastases in a murine breast cancer model (174). Currently, IL-17 specific antibodies are being tested in psoriasis patients, but more preclinical studies are needed before this could also be applied in cancer patients (201).

In humans, G-CSF-mobilized neutrophils display immunosuppressive activity (55). Interestingly, we reported that neutrophil mobilization for granulocyte transfusion purposes silenced the ability of neutrophils to suppress T cell responses (202). The lack of MDSC activity was identified while our findings demonstrated unaltered anti-microbial effector activities, including motility, ROS formation, degranulation and killing of bacteria and fungi (202, 203). This important finding suggests that MDSC activity comprises common effector functions combined with a distinct and unique activity that is (only) involved upon MDSC induction. This finding raises the possibility of using a selective approach in patients to silence immunosuppressive neutrophils and to thereby enhance T cell activity in tumors. Alternatively, proteins of the complement system represent potential targets to block the immunomodulatory and pro-tumor activity of neutrophils, since the importance of C3 activation in T cell suppression by neutrophils from cancer patients was nicely illustrated (58).

#### Targeting Additional Pro-tumor Function of Neutrophils

As described in previous sections, neutrophils rely on several mechanisms to exert their pro-tumorigenic function. Interfering with these downstream tumor-enhancing effects could provide therapeutic benefit. For instance, increased uptake of fatty acids by murine PMN-MDSCs was demonstrated to support their immunosuppressive activity (204). Accordingly, PMN-MDSCs of patients with head and neck, lung, or breast cancer displayed lipid accumulation, along with increased expression of FATP2, a fatty acid transporting protein (205). FATP2 deletion in mice resulted in the loss of the ability to suppress T cell responses leading to a delay in tumor progression. However, most findings from these experimental studies were obtained in mouse models, which by definition cannot be easily extrapolated to human cancer (206).

Other studies have focused on targeting NE, which contributes to cancer progression through enhanced proliferation, invasion and metastasis (31–33, 35–37, 207, 208). Suppression of NE activity by the small molecule sivelestat resulted in reduced tumor growth in murine models of colorectal and prostate cancer (207, 208). The NE inhibitor sivelestat sodium hydrate has been successfully applied in esophageal cancer patients, although this study did not focus on tumor progression (209). Furthermore, NETs may represent a target for reducing the pro-tumor effects of neutrophils since their elimination by the DNA degrading enzyme DNase I is a method that has been established and is tested in clinical trials^1^, though not yet in cancer (210). More clinical studies are necessary to investigate the potential of targeting NE or NETs in human cancer patients. The potential ways to limit pro-tumorigenic activity of neutrophils are summarized in Figure 1; 1–4.

### Promoting Anti-tumor Capacity of Neutrophils

#### Releasing the Off-Switch

Although the above-mentioned approaches to modulate MDSC activity or other pro-tumorigenic effector functions of neutrophils are very promising, redirecting the toxic MDSC activity against T cells toward an anti-tumorigenic role might contribute and steer the effector functions away from neutrophils as suppressor cells to effective tumor-killing in the TME. We have already described the efficacy of neutrophils attacking antibody-opsonized tumor cells, and there by we could speculate that the presence of a mAb could already shift MDSC activity to a certain extent by binding to the Fcγ receptor on their surface and subsequently inducing a cytotoxic response.

Recent research has been focusing on further augmenting the anti-tumor responses of neutrophils, i.e., by trying to switch off the brakes. Neutrophils express a variety of inhibitory receptors on their cell surface (211) providing potential therapeutic targets for checkpoint-blockade therapy. One well-established example of successful checkpoint-blockade on neutrophils is CD47-signal regulatory protein alpha (SIRPα) disruption (212). We and others have already shown the potency of blocking the interaction between CD47 and SIRPα on neutrophils, thereby enhancing the neutrophil’s ADCC capacity against both solid and hematological tumors *in vitro* and *in vivo* (213–215). Antibodies targeting either SIRPα or CD47 have been recently described, showing high efficacy and minimal to moderate toxicity effects (216–218) and a number of clinical trials with these CD47-SIRPα-interfering agents are ongoing^1^. In particular, Hu5F9-G4 against CD47 was tested in combination with rituximab for the treatment of patients with Non-Hodgkin’s lymphoma in a phase 1b clinical trial (219) inducing durable complete responses while no clinically significant toxicity events were observed. At the moment, Hu5F9-G4 is tested in combination with cetuximab for the treatment of colorectal cancers in a phase 1b/2 clinical trial (NCT02953782). Pre-clinical data further showed that blockade of the CD47-SIRPα checkpoint on innate cells eventually activates an anti-tumor response in T cells, bridging the innate with the adaptive immunity (220). Indeed, macrophages were shown to function as APCs and thereby activate the CD8^+^ T cell population while decreasing priming of CD4^+^ T cells after anti-CD47-induced phagocytosis of tumor cells (221). Also DCs are able to contribute to the therapeutic effect of anti-CD47 treatment through cross-priming of CD8^+^ T cells (222). Results from additional future clinical studies will demonstrate whether targeting the CD47-SIRPα axis in a clinical setting indeed will activate both innate as well as adaptive anti-tumor immunity.

Overall, most current studies considered macrophages or NK cells to be the major effector cells in innate checkpoint-blockade antibody therapy (223–225). Given that neutrophils are endowed with similar inhibitory receptors, they can potentially acquire a supporting anti-tumor response. Recent studies have elucidated that Sialic acid-binding immunoglobulin-like lectins or Siglec-blockade can augment the anti-tumor response both by reinvigorating the innate cells and by depleting MDSCs from the TME, thereby unleashing the T cells of the adaptive immune system (180, 226–228). In another perspective, targeting hypersialylation on tumor cells, a trait related to tumor progression and therapy resistance, could also inhibit the Siglec-sialoglycan axis and re-inforce the anti-tumor response (229). The role of Siglec checkpoint-blockade has been highlighted in neutrophils, where research on Siglec-9 or its murine equivalent Siglec-E showed up regulation of the protein receptor in the cytotoxic synapse formation between neutrophils and carcinoma cells, while incubation of neutrophils with anti-Siglec-9 mAbs resulted in significantly increased tumor cell killing by neutrophils (230–233).

Enhancement of the anti-tumor response of neutrophils can also be achieved by targeting signaling partners downstream of the inhibitory receptors. Even better, combination of ligand-receptor interaction disruption and simultaneous blockade of a protein functioning downstream, could have a substantial impact (234, 235). Protein tyrosine kinases (PTKs) and protein tyrosine phosphatases (PTPs) are two important regulators of immune cell responses. As they bind directly to the ITIM motif of the cytoplasmic tails of inhibitory receptors, drugs targeting their function could potentially amplify the neutrophil effector function against tumor cells (236). Despite the above-mentioned promising data on neutrophil effector function, evidence from *in vivo* experiments will shed more light on the complexity of neutrophil anti-tumor response.

#### Augmenting the On-Switch

A different perspective that could redirect pro-tumor and immunosuppressive activity of neutrophils to improve their cytotoxic responses toward cancer in the context of antibody therapy, could be the use of alternative antibody isotypes compared to the classical IgG1. Particularly, IgA antibodies, which specifically bind the IgA Fcα receptor I (FcαRI or CD89) present on cells of the myeloid lineage, including neutrophils (237), are currently considered as a promising approach in immunotherapy against cancer because of their superior ability to induce neutrophil-mediated ADCC. This was reported for a number of tumor-associated antigens such as Ep-CAM for colon carcinoma, HER2/neu for breast carcinoma, EGFR for epithelial, colorectal and renal cell carcinoma, HLA class II, CD20 and CD30 for B-cell lymphoma, and carcinoembryonic antigen as shown in *in vitro* studies (127, 128, 238–242). The induction of such stronger cytotoxic responses upon an IgA engagement of human neutrophils could be explained by the higher avidity of FcαRI which binds bivalently to IgA, and hence recruit more ITAMs to initiate a more robust signaling to activate effector functions (242). Noteworthy, immature neutrophils that were mobilized from the bone marrow after G-CSF treatment triggered a more efficient *ex vivo* tumor cell lysis in the presence of an IgA antibody compared to IgG (244, 245). Based on this we speculate that the use of IgA antibodies could unleash the cytotoxic potential of G-CSF mobilized immature neutrophils in antibody therapy of cancer that otherwise would be immunosuppressive, or even trigger re-polarization of PMN-MDSCs, due to FcαRI constitutive expression (128). In an *in vivo* setting, however, the use of IgA tumor-targeting antibodies is restricted due to the lack of an FcαRI homolog in mice. Nevertheless, the existing genetic engineering techniques have allowed the creation of FcαRI transgenic mouse models, which have been used to confirm the powerful capacity of IgA-mediated tumor killing by myeloid cells in a few studies for EGFR^+^ tumors (246, 247). An important limitation of IgA antibodies *in vivo* as well as in humans is the short half-life compared to that of IgG isotypes (15 h versus 4 days, respectively, in mice, and 5–6 days versus 21 days, respectively, in humans) (237, 246, 248). Attempts to extend the half-life of such promising antibodies or combine them with immune checkpoint blockade therapy are currently being studied (249, 250) and will bring new insights for human application in the near future.

Alternatively, several other approaches to increase ADCC activity of therapeutic antibodies are currently being studied. The majority of these approaches involve glyco- and protein-engineering of IgG1-Fc portions to improve the binding affinities to the activating Fcγ receptors on immune effector cells. On the one hand, it is now firmly established that the glycosylation patterns of the IgG-Fc region are essential for the activation of downstream biological mechanisms of the molecule. Consequently, interfering with such post-translational modifications can drastically influence the effector functions of the immune cells binding to it (137, 251). Specifically, core-fucosylation modifications of the IgG-Fc part are the ones showing a more significant effect (252), although Fc galactosylation and sialylation can have an influence as well (253, 254). In particular, the removal of the core fucose from Fc glycans of IgG1 was shown to increase the binding affinity to FcγRIIIa on NK cells, which resulted in a significant enhancement of ADCC activity for this particular effector cell type (255–257). In the case of neutrophils as effector cells, a reduction of the fucose content of the mAb actually abolished anti-tumor activity instead, indicating that antibody fucosylation differentially impacts cytotoxicity mediated by human NK cells and neutrophils (148). A similar finding was described upon deglycosylation of alemtuzumab (114). These observations may be explained by the fact that human neutrophils only express the decoy receptor FcγRIIIb, which was found to bind with high affinity to low-fucose antibodies, thereby impeding antibody efficacy. A better approach to specifically enhance neutrophil-mediated ADCC responses could be achieved by interfering with the amino acid sequences of the Fc region of the targeting antibody. Specific mutations in this region lead to a higher affinity of anti-EGFR mAbs to the activating FcγRIIa on neutrophils rather than to the decoy FcγRIIIb, which resulted in a restored ADCC activity by purified human neutrophils (257). This approach should be considered to enable the successful development of “next-generation” antibodies when targeting neutrophils as promising effector cells. The most encouraging ways to enhance the anti-tumorigenic activity of neutrophils are depicted in Figure 1; 5–8.

## Concluding Remarks and Future Perspectives

Over time, neutrophils have been identified as important players in cancer, having the unique capacity to either promote or suppress tumor progression. In the present review, we have first provided an overview of such opposing functions of neutrophils that neutrophils can perform. Cancer progression is mediated by neutrophils via several mechanisms, such as the promotion of proliferation, angiogenesis, invasion and metastasis, as well as via suppression of anti-tumor T cell responses. Potential ways to limit the pro-tumor activity of neutrophils include the reduction of neutrophil numbers, or the inhibition of the recruitment or activation of immunosuppressive neutrophils. Conversely, neutrophils can efficiently act as effector cells toward cancer when triggered in the presence of a therapeutic antibody opsonizing the cancer cells, leading to tumor elimination. By releasing the brakes that suppress neutrophils (i.e., interfering with immune checkpoints) or by augmenting the affinity to the opsonizing antibody of interest, the anti-tumorigenic capacities of neutrophils can be significantly enhanced. As research focuses more and more on exploiting neutrophils against cancer, we anticipate that the aforementioned approaches will prove to be highly valuable to suppress the pro-tumor capacities of neutrophils and consequently fully unleash the anti-tumor potential of neutrophils. However, the similarity of these yet distinguished populations, may make the neutrophil-specific targeting difficult to accomplish *in vivo*. The coming years of neutrophil-related research will help understanding neutrophil behavior, while by using new developments we might witness a new era on harnessing neutrophil function against tumor progression.

## Author Contributions

HM and TK are the principle investigators who designed and supervised the work. CF, PM-S, and PB wrote the manuscript. All authors contributed to the article and approved the submitted version.

## Conflict of Interest

The authors declare that the research was conducted in the absence of any commercial or financial relationships that could be construed as a potential conflict of interest.

## Footnotes

1 https://clinicaltrials.gov/

## Abbreviations

ADCC/P: antibody-dependent cellular cytotoxicity/phagocytosis
APC: antigen-presenting cell
CAR: chimeric antigen receptor
CCL: chemokine (C-C) ligand
CCR: C-C chemokine receptor
CDC: complement-dependent cytotoxicity
CGD: chronic granulomatous disease
CLL: chronic lymphocytic leukemia
CSF-1R: colony-stimulating factor-1 receptor
CTLA-4: cytotoxic T-lymphocyte -associated protein 4
CXCR: C-X -C chemokine receptor
DC: dendritic cell
EGFR: epidermal growth factor receptor
ERK: extracellular signal-related kinase
FATP2: fatty acid transport protein 2
Fc γ receptor: IgG Fc receptor
Fc α RI: IgA Fc receptor
FHL-5: familial hemophagocytic lymphohistiocytosis type 5
fMLF: N-formylmethionyl -leucyl-phenylalanine
G-CSF: granulocyte-colony stimulating factor
GM-CSF: granulocyte-macrophage colony-stimulating factor
H_2_O_2_: hydrogen peroxide
HDN: high-density neutrophil
HGF: hepatocyte growth factor
HLA: human leukocyte antigen
ICAM: intercellular adhesion molecule
IFN: interferon
Ig: immunoglobulin
IL: interleukin
IRS-1: insulin receptor substrate 1
ITAM/ITIM: immunoreceptor tyrosine-based activation/inhibitory motif
LAD-1: leukocyte adhesion deficiency type 1
LDN: low-density neutrophil
LOX-1: lectin-type oxidized LDL receptor 1
LPS: lipopolysaccharide
mAb: monoclonal antibody
Mac-1: macrophage-1 antigen
MDSC: myeloid-derived suppressor cell
MHC: major histocompatibility complex
MM: multiple myeloma
MMP-9: matrix metalloproteinase-9
NADPH: nicotinamide adenine dinucleotide phosphate
NE: neutrophil elastase
NET: neutrophil extracellular trap
NF κ B: nuclear factor kappa B
NK: natural killer
NLR: neutrophil-to-lymphocyte ratio
PBMC: peripheral blood mononuclear cell
PD-1: programmed cell death protein 1
PDGFR: platelet-derived growth factor receptor
PD-L1: programmed death-ligand 1
PI3K: phosphoinositide 3-kinase
PMN: polymorphonuclear leukocyte
PTK: protein tyrosine kinase
PTP: protein tyrosine phosphatase
ROS: reactive oxygen species
Siglec: Sialic acid-binding immunoglobulin-like lectin
SIRP α: signal regulatory protein alpha
TAN: tumor-associated neutrophil
TGF- β: transforming growth factor- β
TLR: toll-like receptor
TME: tumor microenvironment
TNF α: tumor necrosis factor α
Treg: regulatory T cell
TRMP2: transient receptor potential cation channel subfamily M member 2
VEGF: vascular endothelial growth factor.
